# Bulbar Palsy Due to Pachymeningitis as an Initial Manifestation of Granulomatosis With Polyangiitis

**DOI:** 10.7759/cureus.70261

**Published:** 2024-09-26

**Authors:** Prem Balaji Reddy Lankapothu, Saranya Chinnadurai, Shrinidhi Bhaskaran, Arun Kumar Bathena, Sharath Chandra Dasi

**Affiliations:** 1 General Medicine, Saveetha Medical College and Hospitals, Saveetha Institute of Medical and Technical Sciences, Saveetha University, Chennai, IND; 2 Rheumatology, Saveetha Medical College and Hospitals, Saveetha Institute of Medical and Technical Sciences, Saveetha University, Chennai, IND

**Keywords:** anti-pr3, bulbar palsy, cranial nerve paralysis, cyclophosphamide methylprednisolone pulse, idiopathic hypertrophic cranial pachymeningitis, mri, mycophenolate mofetil (mmf), wegener’s granulomatosis

## Abstract

Granulomatosis with polyangiitis (GPA), formerly known as Wegener’s granulomatosis, is a rare autoimmune vasculitis that primarily affects small to medium-sized blood vessels, typically involving the respiratory tract and kidneys. However, central nervous system involvement, particularly in the form of pachymeningitis, is an exceptionally rare presentation. This case report details a 55-year-old female who presented with pachymeningitis as the initial manifestation of GPA. The patient exhibited non-specific symptoms, including ear pain, voice changes such as hoarseness of voice, and dysphagia, suggestive of neurological deficits affecting lower cranial nerves IX, X, and XII.

Diagnostic imaging, particularly MRI, revealed characteristic smooth dural thickening and enhancement, suggesting an inflammatory etiology. Laboratory investigations, including positive cytoplasmic-antineutrophil cytoplasmic autoantibody and anti-proteinase 3 antibodies, confirmed the diagnosis of GPA. The patient was treated with high-dose corticosteroids and mycophenolate mofetil, followed by cyclophosphamide due to a relapse, resulting in significant improvement in her condition.

This case underscores the importance of considering GPA in the differential diagnosis of pachymeningitis and highlights the diagnostic challenges posed by its nonspecific symptoms. Early recognition and a multidisciplinary approach are crucial for effective management and prevention of severe neurological complications. The report also emphasizes the need for adherence to treatment regimens to avoid relapses and manage the disease effectively.

## Introduction

Granulomatosis with polyangiitis (GPA), previously known as Wegener’s granulomatosis, is a rare autoimmune disorder characterized by necrotizing granulomatous inflammation and vasculitis affecting small to medium-sized blood vessels [1]. This systemic disease predominantly involves the upper and lower respiratory tracts and kidneys but can affect virtually any organ system [1,2]. The etiologic of GPA is not entirely understood, but it is associated with the presence of antineutrophil cytoplasmic antibodies (ANCAs), specifically those targeting proteinase 3 (PR3-ANCA) [2,3].

While the respiratory and renal systems are most commonly affected, central nervous system (CNS) involvement, although rare, has been increasingly recognized. CNS manifestations can range from ischemic strokes and intracranial hemorrhages to cranial neuropathies and meningeal involvement [4]. Among these, hypertrophic pachymeningitis, characterized by inflammation and thickening of the dura mater, is particularly rare and can lead to various neurological symptoms.

Pachymeningitis in the context of GPA poses significant diagnostic challenges. Its clinical presentation is often non-specific, with symptoms that can overlap with other infectious, inflammatory, and neoplastic conditions. Common symptoms include chronic headaches, cranial nerve palsies, and signs of increased intracranial pressure, which can easily be misattributed to other more prevalent conditions [4-6]. Radiologically, pachymeningitis is identified by the thickening and enhancement of the dura mater on contrast-enhanced MRI [7,8].

This case report presents a unique instance of a 55-year-old female who initially presented with pachymeningitis as the manifestation of GPA. Her symptoms included ear pain, voice changes, dysphagia, significant weight loss, and cranial nerve deficits. Through comprehensive clinical, radiological, and laboratory evaluations, a diagnosis of GPA was established, and appropriate immunosuppressive therapy was initiated, leading to an improvement in her symptoms. This case underscores the importance of considering GPA in the differential diagnosis of pachymeningitis and highlights the necessity of early recognition and treatment to prevent severe neurological complications.

## Case presentation

A 55-year-old female with no comorbidities presented with a six-month history of right ear pain, followed by a two-month history of voice changes, and one month of dysphagia progressive from solids to liquids. She reported symptoms suggestive of possible aspiration such as nasal regurgitation of food and coughing during oral intake, along with a significant weight loss of 10 kg over the past month. Additionally, she experienced slurred speech for two months with a nasal quality and a low-pitched voice with understanding the speech and replying with appropriate answers slurring in nature, able to read and write, worsening dysphagia over the past week, as well as other symptoms such as dysphonia, dysarthria, difficulty in chewing, and intermittent headaches. The patient denied fever, visual disturbances, hearing loss, dizziness, loss of consciousness, seizures, limb weakness, shrugging of shoulders, emotional incontinence, impaired gait, recent trauma, skin rashes, joint pains, abdominal pain, chest pain, or breathing difficulties.

Clinical examination revealed a moderately built and nourished patient, with stable vitals. Physical examination revealed no weakness, ataxia, normal deep tendon reflexes, and normal jaw reflex and bilateral plantar reflex flexor. On cranial nerve examination, cranial nerves I to VIII were normal. Deviation of the tip of the tongue to the right side with reduced movement of the soft palate was suggestive of XIIth and Xth cranial nerve involvement. Both tympanic membranes were intact and clear. Nasal examination revealed a deviated nasal septum to the left. Video laryngoscopy demonstrated laryngeal crepitus, left vocal cord palsy in the median position with compensatory movement of the right vocal cord, and pooling of saliva in the bilateral pyriform sinuses and the post-cricoid region, with an absent gag reflex, diagnosed as bulbar palsy suggestive of the involvement of IX, X, and XII cranial nerves.

**Figure 1:**
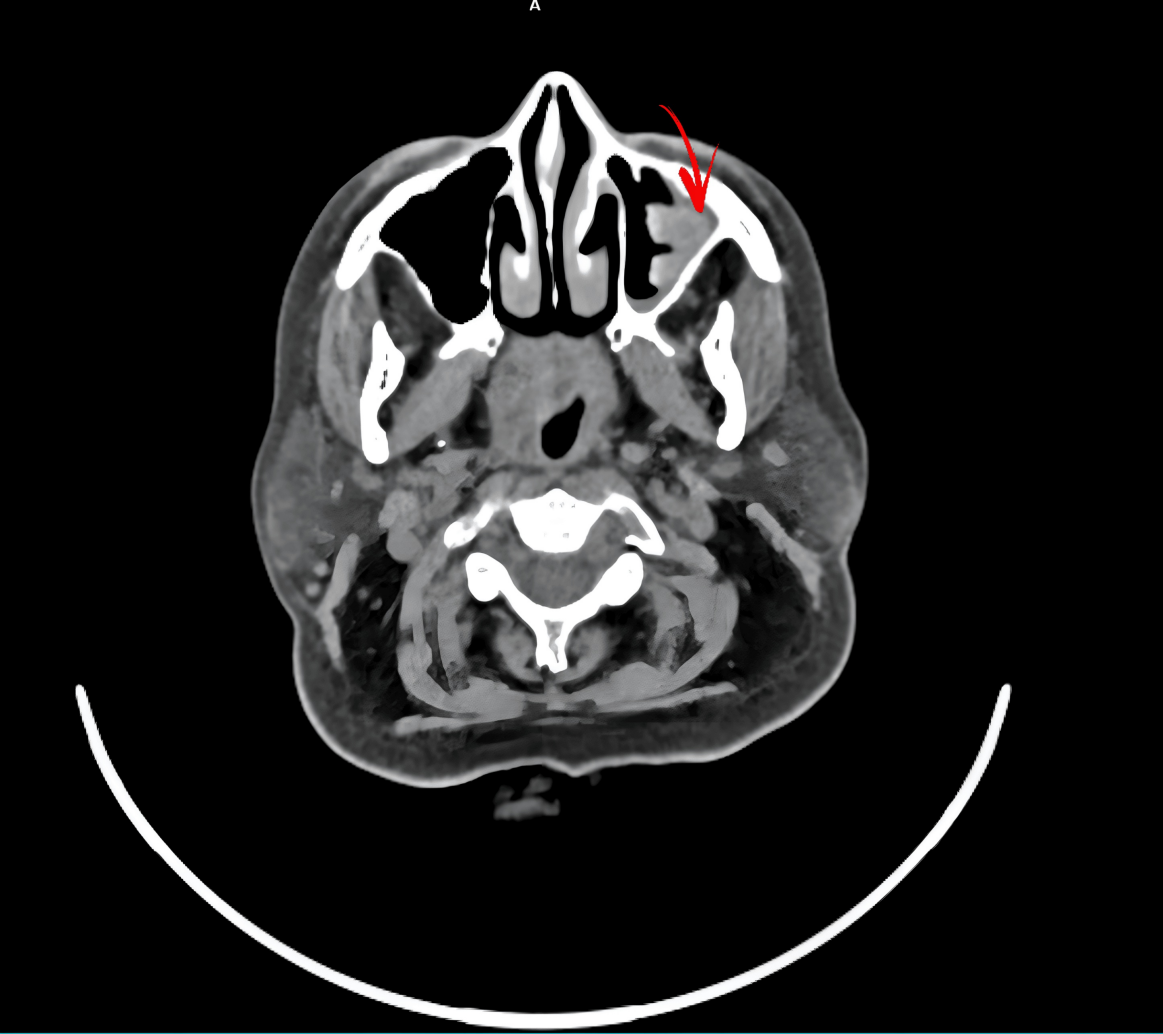
CT of the paranasal sinuses and base of the skull. CT shows moderate irregular mucosal thickening in the left maxillary sinus and mild paucity of right mastoid air cells.

**Figure 2:**
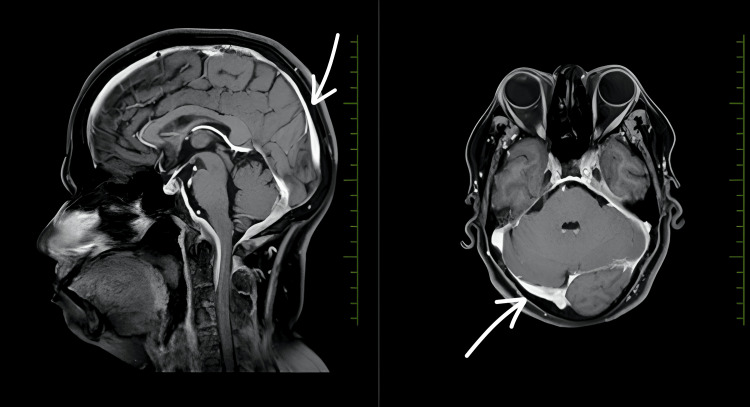
MRI with contrast sagittal and axial view. There is smooth dural thickening and enhancement of pachymeninges along the mid and post-cranial fossa. Anteriorly: Extending from the clivus superiorly to the tectorial membrane and dura of the spinal cord at the level of the C2 vertebra. Posteriorly: Extending along the bilateral tentorium cerebelli (left > right) and falx cerebelli.

**Figure 3:**
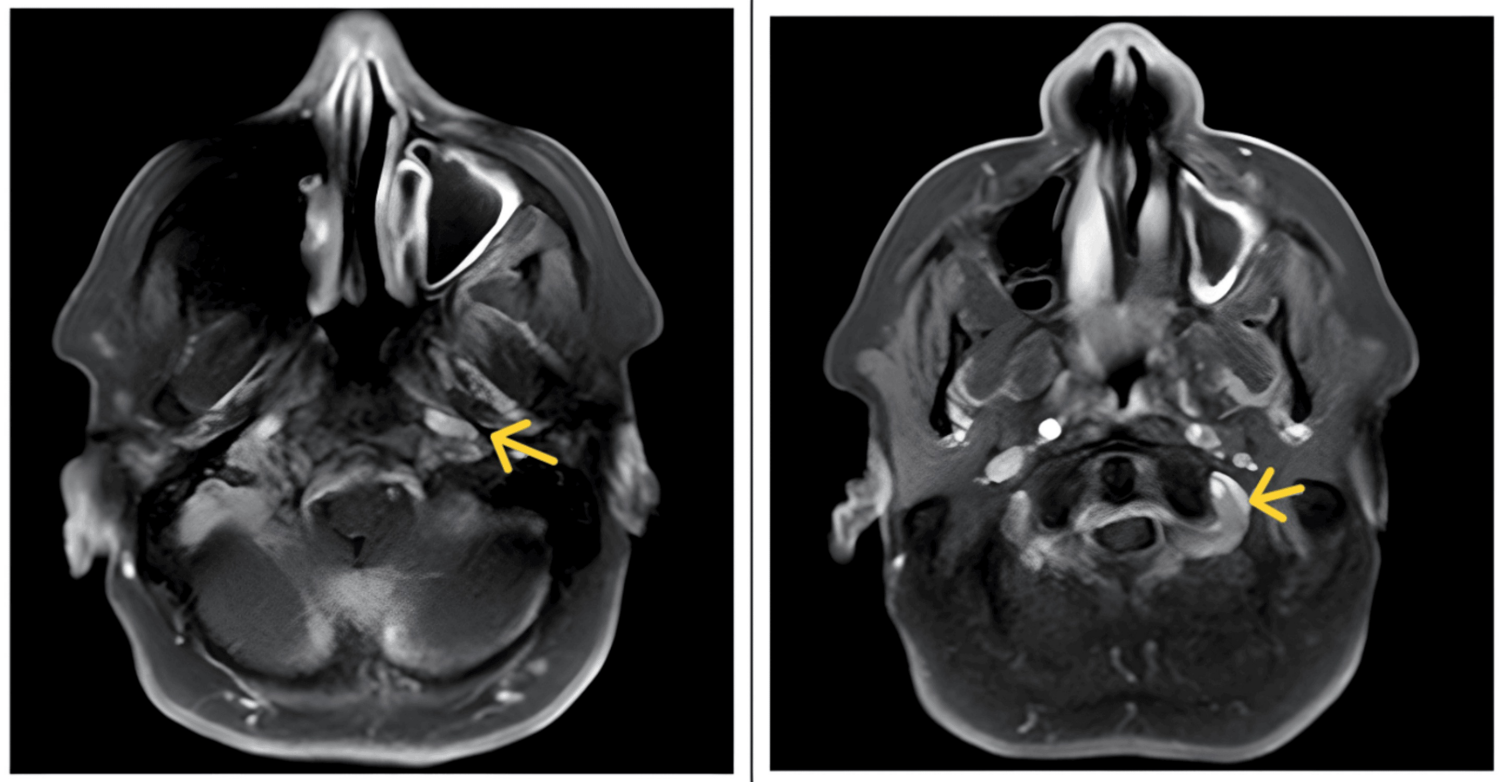
MRI with contrast axial view. Mild thickening and enhancement of bilateral IXth, Xth, and XIIth cranial nerves at the level of the medulla to the jugular foramen and XIIth nerve at the level of the medulla.

**Figure 4:**
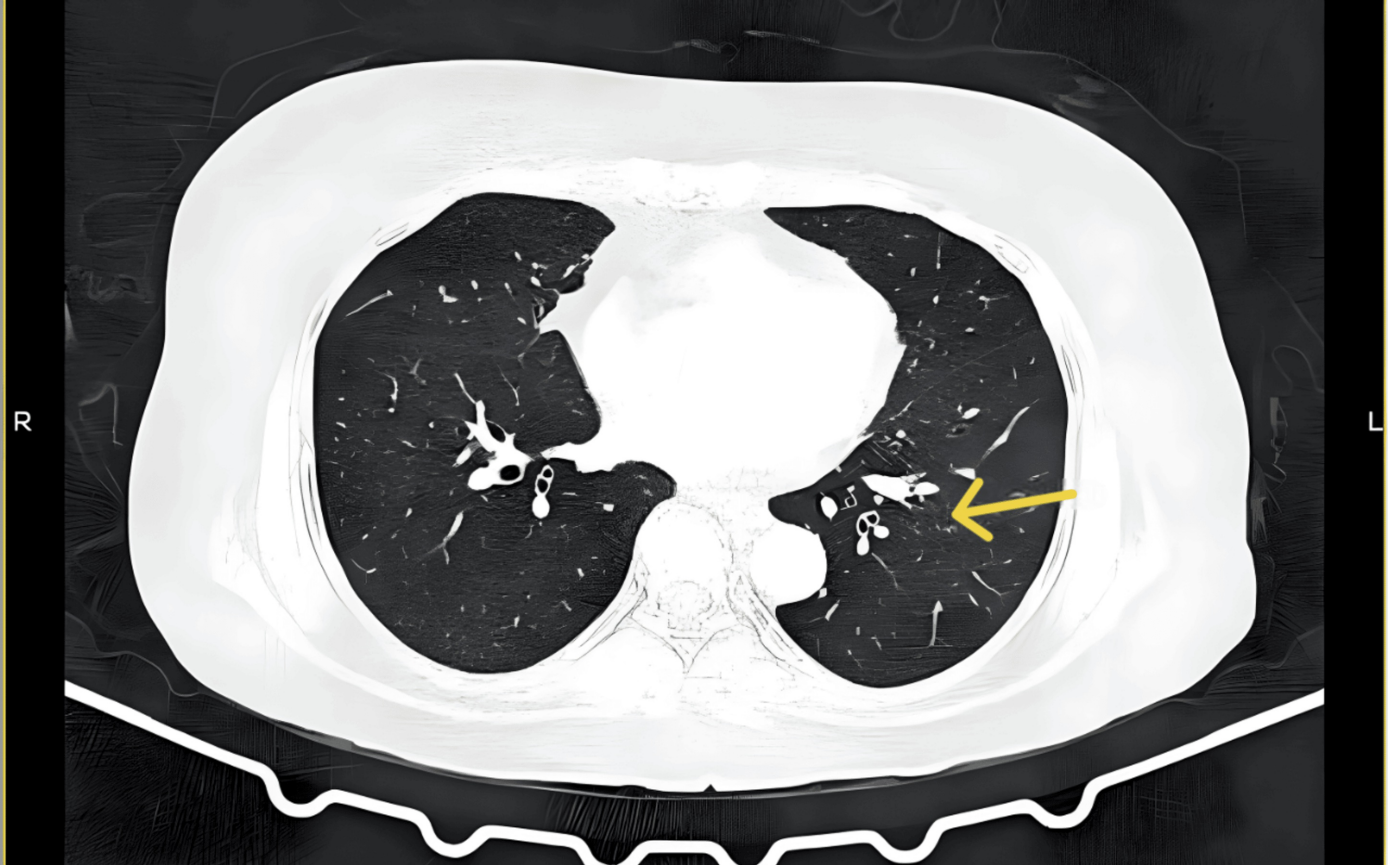
CT of the thorax. CT of the thorax shows few fibrotic strands with subtle ground-glass opacity in the left lower lobe, with normal trachea, bronchi, heart, and mediastinal vessels.

CT of the paranasal sinuses and skull base revealed moderate irregular mucosal thickening in the left maxillary sinus, mild paucity of right mastoid air cells, and otherwise normal sinuses (Figure 1). MRI of the brain with contrast showed smooth dural thickening and enhancement of pachymeninges along the mid and posterior cranial fossa, extending from the clivus to the dura of the spinal cord at the level of the C2 vertebra, and thickening and enhancement of bilateral IXth, Xth, and XIth cranial nerves at the level of the medulla to the jugular foramen, and the XIIth nerve at the level of the medulla (Figures 2, 3). CT of the thorax showed few fibrotic strands with subtle ground-glass opacity in the left lower lobe, with normal trachea, bronchi, heart, and mediastinal vessels (Figure 4).

Laboratory investigations including complete blood count, renal and liver function tests, and urinalysis were normal. Cerebrospinal fluid (CSF) analysis revealed 29 cells/mm^3^ (neutrophils, 4%; lymphocytes, 96%), normal glucose, normal protein, and clear cytology with red blood cells. GeneXpert testing and culture were negative. Serum studies showed normal serum immunoglobulin G4 and angiotensin-converting enzyme levels. C-reactive protein level was 22 mg/mL, erythrocyte sedimentation rate was 24 mm/hour, C3 was 80 mg/dL, and C4 was 40 mg/dL. Serology including human immunodeficiency virus, hepatitis C virus, and hepatitis B surface antigen were negative. The autoimmune panel revealed c-ANCA positivity, p-ANCA negativity, antinuclear antibody immunoblot anti-Smith positivity, and anti-PR3 enzyme-linked immunosorbent assay at 80 IU/L (Table 1). Meningeal biopsy was advised but the patient deferred the procedure. European Vasculitis Study Group (EUVAS), Birmingham Vasculitis Activity Score modified for Wegener’s Granulomatosis (BVAS/WG), and Vasculitis Damage Index (VDI) scores were calculated to assess disease activity. The BVAS/WG score was 8, and the VDI score was 4 in this patient. These scores indicated significant disease activity and chronic damage in this patient, emphasizing the severity of the condition and the need for aggressive and sustained immunosuppressive therapy. Regular monitoring and treatment adjustment were essential to manage the disease effectively and prevent further complications.

**Table 1:** Lab investigations. CSF: cerebrospinal fluid; CBC: complete blood count; RFT: renal function test; LFT: liver function test; CRP: C-reactive protein; ESR: erythrocyte sedimentation rate; C3: complement 3; C4: complement 4; c-ANCA: cytoplasmic antineutrophil cytoplasmic antibodies; p-ANCA: perinuclear antineutrophil cytoplasmic antibodies; ANA: antinuclear antibodies; ACE: angiotensin-converting enzyme; IgG4: immunoglobulin G4; PR3: proteinase 3; ELISA: enzyme-linked immunosorbent assay; RBC: red blood cells; HIV: human immunodeficiency virus; HCV: hepatitis C virus; HBsAg: hepatitis B surface antigen

Investigation	Results	Reference range
CSF analysis	29 cells/mm^3^	
CSF cells	29 cells/mm^3^	0-5 cells/mm3
CSF neutrophils	4%	<10%
CSF lymphocytes	96%	70–80%
CSF glucose	56	40–70 mg/dL
CSF protein	19	15–45 mg/dL
CSF cytology	Clear	Clear
CSF RBCs	Seen	Absent
CSF GeneXpert	Negative	Negative
CSF culture	Negative	Negative
Serum IgG4	Normal	<135 mg/dL
Serum ACE	Normal	8–53 U/L
CRP	22 mg/L	<5 mg/L
ESR	24 mm/hour	0–20 mm/hour
C3	80 mg/dL	90–180 mg/dL
C4	40 mg/dL	10–40 mg/dL
c-ANCA	Positive	Negative
p-ANCA	Negative	Negative
ANA immunoblot	Anti-Smith positivity	Negative
Anti-PR3 ELISA	80 IU/L	<20 IU/L
HIV	Non-reactive	
HCV	Non-reactive	
HBsAg	Non-reactive	
Glycosylated hemoglobin	5.40%	<5.70%

The patient was initially started on intravenous (IV) methylprednisolone (1 g daily for three days as pulse therapy), along with speech and swallowing therapy. She was then transitioned to oral steroids at 1 mg/kg, with a tapering dose, and mycophenolate mofetil (MMF) 500 mg once daily. Despite some initial improvement, she discontinued her medications for two weeks, leading to a worsening of symptoms. Subsequently, treatment was initiated with a stat dose of IV cyclophosphamide (1 g), followed by pulse therapy at 10 mg/kg once monthly, along with a tapering course of oral steroids and MMF 500 mg. Recently, she received her fifth cycle of cyclophosphamide (750 mg), resulting in improvement, including the ability to swallow semi-solid foods. Although her tongue movements improved, slurred speech persisted. Repeat video laryngoscope showed decreased pooling of saliva and other contents. She remains on regular follow-up, showing mild improvement, and further cyclophosphamide therapy is still required. Additionally, the patient continues to require regular speech and swallowing therapy.

## Discussion

This case report presents a unique and diagnostically challenging case of GPA, where pachymeningitis was the initial manifestation. GPA, a rare but severe multisystem autoimmune vasculitis, is characterized by granulomatous inflammation and necrotizing vasculitis primarily affecting small to medium-sized blood vessels [1,2]. While GPA predominantly involves the respiratory tract and kidneys, the CNS can be affected, albeit less commonly. Pachymeningitis, which involves inflammation and thickening of the dura mater, is an exceptionally rare presentation of GPA, making the diagnosis complex and often delayed.

In this case, the patient initially presented with non-specific symptoms such as ear pain, voice changes, and dysphagia, which gradually progressed to more severe neurological manifestations, including significant weight loss, slurred speech, dysphonia, dysarthria, and intermittent headaches. The involvement of cranial nerves, particularly the left vocal cord palsy, and the absence of a gag reflex (Bulbar palsy), were critical in raising suspicion of pachymeningitis [4-6]. However, the presence of chronic headaches served as a crucial indicator of possible CNS involvement [4], warranting further investigation.

The diagnostic process in GPA-related pachymeningitis is often challenging due to the overlap of symptoms with other more common conditions, such as infections, neoplasms, and other inflammatory diseases [3-5]. In this case, imaging studies played a pivotal role in narrowing down the differential diagnosis. MRI findings of smooth dural thickening and enhancement, extending from the clivus to the dura of the spinal cord at the C2 vertebra level, were characteristic of pachymeningitis and strongly suggested an inflammatory etiology [7,8]. The involvement of multiple cranial nerves further supported this diagnosis. Importantly, the absence of typical signs of infection or neoplasm on imaging helped focus the differential diagnosis on autoimmune causes, including GPA.

Laboratory investigations further substantiated the diagnosis. The CSF analysis showed mild lymphocytic pleocytosis with normal glucose and protein levels, indicative of an inflammatory process rather than an infectious one. The detection of positive c-ANCA and anti-PR3 antibodies was crucial in confirming GPA, as c-ANCA with PR3 specificity is highly specific for this disease and correlates closely with disease activity [2,9]. The positive ANA immunoblot for anti-Smith antibodies, though more commonly associated with systemic lupus erythematosus, also suggested the possibility of an overlap syndrome, adding another layer of complexity to the diagnosis.

The management of GPA-related pachymeningitis requires a carefully tailored approach. In this case, the patient was initially treated with IV steroids to quickly reduce inflammation, followed by a maintenance regimen of oral steroids and MMF 500 mg once daily, a steroid-sparing agent [9,10]. The initial response to treatment was positive; however, the patient experienced a relapse after discontinuing her medication for two weeks, which led to a worsening of symptoms. This underscores the importance of adherence to the prescribed treatment regimen and the need for close monitoring to prevent relapses. The subsequent administration of IV cyclophosphamide pulse therapy monthly once 10 mg/kg dose, a potent immunosuppressant, was necessary to regain control of the disease and achieve symptom improvement in swallowing; however, mild symptoms of slurring of speech persistet. The patient remains on follow-up and the fifth cycle of injection cyclophosphamide 750 mg [9,10].

The case also highlights a broader challenge in managing GPA-related pachymeningitis: the lack of standardized treatment protocols, especially when neurological symptoms are involved. While high-dose glucocorticoids and immunosuppressants are the cornerstone of treatment, the introduction of newer therapies, such as Avacopan, which acts as a selective C5a receptor antagonist, offers promising alternatives. Avacopan, used in combination with rituximab or cyclophosphamide, has been shown to be effective in reducing glucocorticoid use and managing disease activity in GPA as per European Alliance of Associations for Rheumatology guidelines [11]. However, its specific role in treating pachymeningitis remains to be fully understood, and further research is needed to establish its efficacy in this context.

The prognosis for patients with GPA, particularly those with CNS involvement such as pachymeningitis, depends significantly on the timeliness of diagnosis and initiation of appropriate therapy. Delayed treatment can lead to irreversible neurological damage and other severe complications [9,10]. This case underscores the importance of a multidisciplinary approach in the management of such complex conditions, involving rheumatologists, neurologists, radiologists, and other specialists, to ensure comprehensive care. Regular follow-up, with ongoing clinical assessments and laboratory monitoring, is essential to prevent relapses and ensure the best possible outcomes for the patient.

This case adds to the growing body of literature on the diverse presentations of GPA and highlights the necessity of considering autoimmune vasculitis in patients presenting with atypical neurological symptoms. The integration of clinical findings with advanced diagnostic modalities, such as MRI and specific antibody testing, is crucial for timely and accurate diagnosis. Furthermore, this case illustrates the importance of sustained and carefully managed immunosuppressive therapy in preventing severe complications and managing GPA-related pachymeningitis effectively.

## Conclusions

This case highlights the importance of considering pachymeningitis as a possible early sign of granulomatosis with polyangiitis (GPA), an autoimmune disorder with potentially severe outcomes. Accurate diagnosis relies on a combination of clinical evaluation, advanced imaging, and specific laboratory tests like c-ANCA and anti-PR3 antibodies. Early recognition and prompt treatment with immunosuppressive therapy are crucial to prevent serious complications, especially neurological ones.

The successful management of GPA-related pachymeningitis requires a coordinated approach involving multiple medical specialties. This case also emphasizes the necessity of adherence to treatment regimens to avoid relapse and disease progression. As our understanding of GPA continues to evolve, ongoing research and clinical awareness are vital to improving patient outcomes and managing the disease effectively.

## References

[1] Higuera-Ortiz V, Reynoso A, Ruiz N, Delgado-Hernández RD, Gómez-Garza G, Flores-Suárez LF (2017). Pachymeningitis in granulomatosis with polyangiitis: case series with earlier onset in younger patients and literature review. Clin Rheumatol.

[2] Koening CL, von Hennigs I (2021). Antineutrophil cytoplasmic antibody (ANCA) vasculitis: pathophysiology, diagnosis, and the evolving treatment landscape. Am J Manag Care.

[3] Scott DG, Watts RA (2013). Epidemiology and clinical features of systemic vasculitis. Clin Exp Nephrol.

[4] Holle JU, Gross WL (2011). Neurological involvement in Wegener's granulomatosis. Curr Opin Rheumatol.

[5] Nowack R, Wachtler P, Kunz J, Rasmussen N (2009). Cranial nerve palsy in Wegener's granulomatosis--lessons from clinical cases. J Neurol.

[6] Ahmed SV, Chandra S (2013). Widespread cranial nerve palsies while on cyclophosphamide therapy: a very rare manifestation of Wegener's granulomatosis (granulomatosis with polyangitis). BMJ Case Rep.

[7] Abdel Razek AA, Alvarez H, Bagg S, Refaat S, Castillo M (2014). Imaging spectrum of CNS vasculitis. Radiographics.

[8] Antony J, Hacking C, Jeffree RL (2015). Pachymeningeal enhancement-a comprehensive review of literature. Neurosurg Rev.

[9] Alberici F, Tedesco M, Popov T, Balcells-Oliver M, Mescia F (2024). Treatment goals in ANCA-associated vasculitis: defining success in a new era. Front Immunol.

[10] Wallace ZS, Miloslavsky EM (2020). Management of ANCA associated vasculitis. BMJ.

[11] Hellmich B, Sanchez-Alamo B, Schirmer JH (2024). EULAR recommendations for the management of ANCA-associated vasculitis: 2022 update. Ann Rheum Dis.

